# Respiratory Exposure to Highly Fluorinated Chemicals via Application of Ski Wax and Related Health Effects

**DOI:** 10.1007/s40572-023-00425-4

**Published:** 2024-01-13

**Authors:** Kathryn A. Crawford, Nicola Hartmann

**Affiliations:** 1 Environmental Studies Program, Middlebury College, 276 Bicentennial Way, Middlebury, VT 05753 USA; 2 Middlebury College, Middlebury, VT USA

**Keywords:** Per- and polyfluoroalkyl substances (PFAS), Ski wax, Acute respiratory health, Body burden, Airborne PFAS, Particulate matter

## Abstract

**Purpose:**

Waxes containing per- and polyfluoroalkyl substances (PFAS) are applied to the base of skis and snowboards (“skis”) to reduce friction with the snow surface and improve glide. PFAS exposure can adversely impact cardiometabolic, thyroid, liver, kidney, reproductive, and immune health and are associated with increased risk of certain cancers. In the present review, we summarize the state of the science on PFAS exposure from fluorinated ski wax use, including acute respiratory health effects and PFAS concentrations in biological and environmental media collected from ski waxing settings.

**Recent Findings:**

Perfluoroalkyl carboxylic acid (PFCA) concentrations in serum and air collected from professional wax technicians and the rooms where waxes are applied are among the highest of any occupation investigated to date, including the fluorochemical industry. High airborne concentrations of fluorotelomer alcohols contribute to high body burdens of certain PFCAs among ski waxers.

**Summary:**

Fluorinated ski waxes are a significant source of PFAS exposure for people waxing skis and/or spending time in areas where waxing occurs. We highlight recommendations for future research, policy, and technologies needed to address PFAS exposures from fluorinated wax use.

## Introduction

Among the diverse applications of per- and polyfluoroalkyl substances (PFAS) is their use in wax products applied to ski and snowboard (collectively referred to as “ski”) bases. Ski performance reflects an interaction between the ski, skier, and snow for recreational and competitive skiers, alike [1]. Across all levels of sport, modifying the ski base is an attractive way to improve ski performance since it attenuates the meteorologically dependent effects of snow conditions on performance. Depending on ski technique, waxes may either be used to improve glide (skate and classic cross country skiing, alpine skiing, and snowboarding) or grip (classic cross country skiing).

Contemporary wax formulations are proprietary, and details are rarely known by consumers. However, many waxes contain a hydrocarbon substrate with different additives to optimize performance under variable snow conditions. PFAS have were the most common performance enhancing additives used in ski waxes from the 1990s through the early 2020s because their surfactant properties reduce friction with the snow surface, thereby improving glide [2–4]. PFAS are a large class of synthetic, organic chemicals that have been widely used in industrial and manufacturing processes and consumer products since the 1940s [5, 6]. Epidemiologic and toxicological studies have identified a range of detrimental human health effects associated with exposure to certain PFAS, particularly perfluoroalkyl acids: elevated cholesterol, altered immune and thyroid function, liver disease, kidney disease, adverse reproductive and developmental outcomes, and cancer [7, 8]. Exposure to PFAS is widespread in the general population and is thought to occur predominantly through ingestion (e.g., diet and drinking water); however, inhalation is recognized as an understudied route of exposure in both general public and occupational settings [9, 10].

**Fig. 1:**
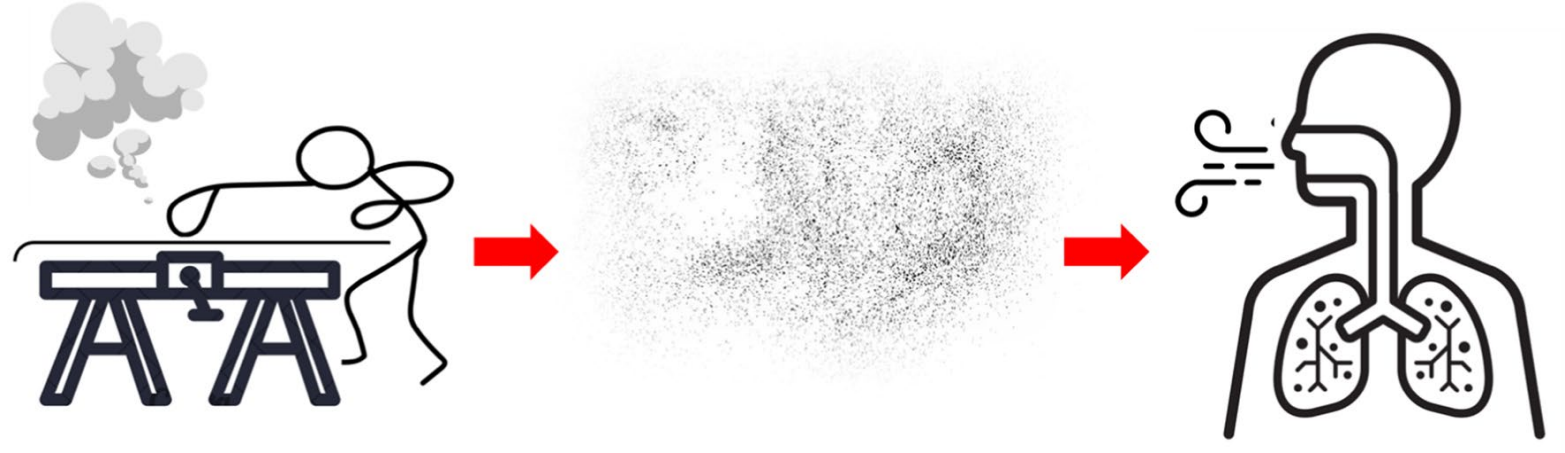
A schematic depicting the exposure pathway of PFAS in fluorinated “fluoro” ski waxes. Once in the lungs, PFAS may enter systemic circulation and be distributed throughout the body

The wax application process has been described previously (e.g., [11] and is summarized visually in Fig. 1. Briefly, waxes are applied to the base of skis using heat — typically from an iron set at > 120 °C, which causes waxes to melt and/or evaporate. For some waxes, the friction generated by vigorous corking is also used to heat waxes into the base of skis. Excess wax is then removed from the base of skis using scrapers and brushes. Heating waxes releases volatile organic compounds and aerosolized particulate matter (PM) into the air where waxing occurs. Mechanically removing excess wax also generates PM. A plume of airborne material is often visible in waxing work spaces [12]. While waxing, the waxer is typically stooped over the ski, leading to direct inhalation exposure of volatiles, aerosols, and PM [13, 14]. Both wax additives and PM may pose human health risks. Furthermore, PFAS from fluorinated waxes are known to contaminate environmental media near ski venues [15–18].

In the present narrative review, we summarize the state of the science on PFAS exposure from fluorinated ski wax use, including PFAS concentrations in biological and environmental media collected from ski waxing settings and acute respiratory health effects. The exact number of people directly impacted by PFAS in fluorinated ski waxes is unknown; however, > 10,000 people in both Sweden [13] and Finland [19] are estimated to be occupationally exposed to ski waxing. In the USA, there are approximately 80,000 people employed within the ski industry as a whole [20] and approximately 7500 registered ski and snowboard coaches. Individuals who ski competitively or recreationally also may own and use fluorinated waxes [21]. Understanding the environmental health impacts of PFAS exposure within this highly exposed population is important for reducing skiers’ PFAS exposure and, more broadly, contributing to knowledge about PFAS exposure via inhalation.

## Methods

We conducted a search for exposure science and epidemiologic literature characterizing PFAS from fluorinated ski waxes in environmental media and human biospecimens, as well as health consequences of these exposures. We identified relevant literature by searching PubMed, Web of Science, and Google Scholar using key words including ski wax, PFAS, inhalation, exposure, and health. Primarily, we focused on literature reporting: (1) PFAS concentrations measured in environmental media collected in or around ski waxing work spaces, (2) PFAS concentrations measured in biospecimens collected from people who apply ski waxes, and (3) adverse health effects attributed to ski wax application. Although beyond the scope of this review, we acknowledge that other environmental health risks besides PFAS exposure may be associated ski waxing.

## PFAS in Ski Wax

Fluorinated waxes are marketed as “fluoros” to advertise their performance-enhancing properties and justify the high cost of these products relative to hydrocarbon-based alternatives. Hundreds of fluoros exist and can be divided into solid, powder, and liquid forms. These waxes contain up to 100% PFAS by mass comprised of perfluorocarboxylic acids (PFCAs), fluoroalkanes, and emerging compounds such as GenX [3, 13, 17, 19, 22, 23]. Fluorinated waxes are classified as low, high, and pure fluoros depending on relative PFAS concentration, though no standardized thresholds for PFAS concentration exist. Global fluorinated wax production was previously estimated to be on the order of several tons annually [17].

### Airborne PFAS From Ski Waxing

Highly elevated body burdens of PFAS, measured as blood PFAS concentration, in ski wax technicians compared to other occupations and the general population [11, 24] are likely due to direct inhalation of vast amounts of airborne PFAS generated during the waxing process. As fluorinated waxes gained popularity in the 1990s, literature emerged documenting the release of volatiles, aerosols, and PM containing PFAS into air during the waxing process. Early investigations focused on PM and inorganic fluorine [13, 25, 26]. Subsequent research focusing on occupational exposures to PFAS from ski waxing at elite levels of competition has significantly expanded knowledge of airborne exposures to PFAS from fluorinated wax use. Collectively, findings from these studies show that use of fluorinated ski waxes can lead to some of the most severe airborne PFAS concentrations of any occupation studied to date [10].

Area and personal air samples collected from waxing rooms and waxers’ breathing zones, respectively, of World Cup wax technicians were analyzed for PFAS [11, 14, 27]. Over the course of an 8-h workday, PFAS concentrations in area air samples were highest in the inhalable size fraction of aerosols [11, 14, 27]. PFCAs with carbon chain lengths from C_4_ to C_14_ dominate the PFAS profiles in air samples collected from ski waxing environments [11, 14, 27, 28]. The highest reported concentrations were for perfluorohexanoic acid (PFHxA; mean, range 99, 1.39–333 μg/m^3^), perfluorododecanoic acid (PFDoDA; 26.5, 0.93–78.3 μg/m^3^) and perfluorooctanoic acid (PFOA; 16.0, 2.11–52.8 μg/m^3^) [14]. PFCA concentrations were lower in Freberg et al. [11], likely due to differences in sampling and analytical methods. In personal air samples collected from waxers’ breathing zones, PFAS profiles are similar to area samples, though concentrations tended to be higher [14]. In a follow-up study, PFAS comprised up to 50% by mass of the total particulate sample [28] and ironing pure fluoro powders produced the highest PM concentrations [12]. Sulfonic acids were detected in very few air samples, which is consistent with findings that show sulfonic acids are not common constituents of waxes and/or contributors to wax-related exposures.

In air samples targeting combined gaseous and particle phases, fluorotelomer alcohols (FTOHs), notably 8:2 FTOH and 6:2 FTOH, comprised the majority of total PFAS in both area and personal air samples with concentrations up to 997 μg/m^3^, representing up to 99% of ΣPFAS by mass [14, 27]. FTOHs are classified as precursor PFAS because they can be biotransformed to compounds such as PFOA and PFNA in humans and animal models [7]. FTOHs were not detected in aerosol samples indicating that they do not absorb well to this fraction of airborne material.

High concentrations of airborne PFAS reported in these studies reflect the potential for extreme exposure to PFAS among people who wax skis or spend time in areas where waxing occurs. Inhalation has been a less well characterized route of exposure to PFAS compared to diet, so fluorinated ski wax application also offers important insights into health impacts and PFAS body burdens from these exposures.

### Acute Respiratory Health Effects From Exposure to PFAS in Ski Waxes

Knowledge of environmental health risks associated with exposure to fluorinated ski waxes emerged in the early 1990s with reports of acute pulmonary injury, decreased lung diffusion capacity, severe dyspnea, as well as complaints of rhinitis, coughing, and breathlessness after ski waxing [13, 25, 29]. The earliest report of wax-related impacts to human health appeared in a 1990 case report published in the Journal of the Norwegian Medical Association describing the development of polymer-fume fever (“Teflon flu”) and pulmonary edema in a patient who smoked cigarettes that had been contaminated with a pure fluoro wax called *Cera F* [30]. Similar symptoms were reported in a man who waxed skis with fluorinated wax for a group of ski racers [29]. Polymer-fume fever, also referred to as Teflon flu, is caused by inhalation of byproducts of thermal degradation of organofluorine compounds and manifests clinically as flu-like symptoms: fever, shivering, throat soreness, chest tightness, and coughing [31, 32].

Exposure to fluorinated ski wax has also been associated with damage to pulmonary function. Carbon monoxide diffusion capacity was reduced for at least 24 h among a small cohort of people (*n* = 5) who applied *Cera F* for an hour [33]. In a study thought to be more representative of what someone waxing skis for personal use might experience, carbon monoxide diffusion capacity was only reduced for a period of several hours after waxing [25].

### PFAS Biomonitoring Among Ski Waxers

Longer-term impacts — respiratory or otherwise — of fluorinated wax exposure have not been well characterized. However, biomonitoring data reveal that professional ski wax technicians have among the highest known PFAS body burdens of any occupation studied to date [11, 24, 27, 34]. Specifically, serum PFAS concentrations among ski wax technicians are similar to or higher than fluorochemical plant workers, firefighters, and people living in communities with contaminated drinking water [34]. Nilsson et al. [24] reported a mean PFOA concentration of 112 (range 4.8–535) ng/mL in whole blood collected from professional wax technicians (*n* = 8) [24, 27]. Freberg et al. [11] also reported elevated PFOA concentrations in serum (mean, range 53, 15–174 ng/mL; *n* = 13); however, these levels tend to be lower than Nilsson et al. [24]. Since PFAS have a high binding affinity to serum albumin, a factor of 2:1 is typically applied when comparing serum:whole blood PFAS concentrations to account for their preferential accumulation in serum [35].

PFCAs with carbon chain lengths of C_4_ and longer accounted for the majority of PFAS detected in wax technicians’ blood [11, 24, 36]. Concentrations of PFOA and PFNA were detected at levels that are 45 and 300 times higher than the general population, respectively [11, 24, 36]. Years of employment as a wax technician strongly predicts serum PFCA concentrations in longitudinal studies [11, 24, 36]. PFAS concentrations tended to increase in blood over the course of a ski season and peak after the ski season ends [24, 27, 36]. This finding suggests either a physiological delay in the absorption and distribution of PFCAs within the body or biotransformation of PFAS precursors (e.g., FTOHs) to terminal end products such as PFOA and PFNA [27]. Given airborne FTOH concentrations found in waxing work spaces, follow-up studies investigated biotransformation of PFAS precursors to terminal end products in humans, providing strong evidence for this process [36, 37].

Collectively, these studies confirm that very high airborne PFAS concentrations in waxing work spaces and waxers’ breathing zones translate to highly elevated PFAS body burdens among exposed individuals. Levels of fluorinated sulfonic acids tend to be relatively similar between wax technicians and the general public and were not correlated with years of employment [11, 24]. When taken together with knowledge of fluorinated wax formulations [3, 13, 17, 19, 22, 23], these studies strongly implicate ski waxing as the source of elevated PFCAs among these workers.

## Additional Environmental Health Concerns from Ski Waxing

Although beyond the scope of this review, it is worth noting that ski waxing poses additional environmental health risks to people in the vicinity. The process of heating fluorinated waxes into the base of skis releases PM and other volatile organic compounds into the air in waxing work spaces [19]. Both PM and other volatile organic compounds may pose health risks distinct from those associated with PFAS.

PM is generated directly through pyrolysis of waxes during application as well as the removal of excess wax. Higher wax iron temperatures increase pyrolytic impact on ski waxes, creating more visible smoke during the waxing process [13]. Additionally, PM can form indirectly when volatile organic compounds produced during pyrolysis react with other atmospheric constituents and/or recondense [19, 26]. PM generated by ski waxing tends to be dominated by ultrafine and fine particles [13, 14, 19, 26], which can distribute more deeply into the lung and enter systemic circulation, posing risks to respiratory health and distal organs. Research into the effects of PM exposure from ski waxing on pulmonary and immune function have shown adverse physiologic impacts, including localized and systemic inflammation [38].

Ski waxing may also be the source of exposure to additional chemistries added to ski waxes for performance enhancing purposes. Silicone-based compounds, graphite, and some metals (e.g., molybdenum, gallium) are known to be used in certain wax formulations [13, 21]. Lead, zinc, and iron have also be detected in ski waxes [19].

## Conclusions, Recommendations, and Future Directions

Fluorinated ski wax may be a significant source of PFAS exposure for people who personally wax skis and/or who spend time in spaces where waxing occurs. Previous research has focused on occupational high exposure scenarios, but other exposure scenarios are imaginable, including: the low-wage seasonal employee working in the ski tuning shop at a ski resort, or the child of someone who waxes skis in their garage for personal use, or the person who wants to ski faster but is not familiar with wax chemistry. In recent years, awareness of the impacts of PFAS on human health and the environment has grown and this knowledge has led to meaningful steps to reduce people’s exposure to PFAS from fluorinated waxes. Institutional controls (e.g., ventilation) and personal protective equipment are now more common while waxing, especially at high-level competitions. Individual ski areas, organizations, communities, and nations have restricted fluorinated wax use to varying degrees since 2018 [15, 21, 39–42]. The International Ski Federation (FIS) and International Biathlon Union (IBU) banned fluorinated wax use in all competitions within their respective purviews as of the 2023–2024 ski season, a process which was postponed multiple years due to technical delays in developing rapid testing for policy enforcement [43–45]. Furthermore, a growing number of policies restrict the use, sale, and manufacture of PFAS-containing products, including waxes [46, 47].

Nevertheless, risks of adverse impacts from fluorinated ski wax on human health and the environment remain. Our research indicates that fluorinated wax use has far outpaced utilization of exposure reduction strategies such as ventilation or respiratory personal protective equipment [21]. The long biological half-lives of PFAS, especially PFCAs found in ski waxes and wax technicians, mean body burdens accumulated from previous exposures will remain within individuals for many years after exposure ends [7]. Furthermore, PFAS have very long environmental half-lives [48, 49], and residual contamination in ski waxing areas will serve as an ongoing source of exposure. No guidelines currently exist for PFAS remediation methods or standards, presenting a challenge for parties interested in remediating ski waxing spaces. People across all levels of sport still have fluorinated ski wax and given its performance enhancing qualities may be inclined to continue using these waxes in non-regulated settings or do so unknowingly. Even if all fluorinated wax manufacture ceases and voluntary take-back programs [50] successfully collect all fluoros that remain in skiers’ personal possession, robust disposal solutions for PFAS-containing waste remain elusive [51]. Currently, disposal options tend to transfer PFAS from one medium to another and often involve transport of PFAS mass contained in these media to new locations, thereby presenting a cyclical problem [51]. Because waste disposal facilities are disproportionately located near marginalized communities [52, 53], efforts by the ski industry to disentangle itself from a long history of fluorinated wax use should be carefully considered for potential negative consequences.

To address ongoing human health and environmental risks from PFAS in fluorinated waxes, investment in additional research and the development of policies, guidelines, and technologies are needed:

- Research to characterize latent health impacts from exposure to PFAS via inhalation.
- Research to better characterize the potential for dermal absorption of PFAS.
- Engagement with the ski and snowboard community to provided education about ongoing environmental health risks from fluorinated waxes and exposure reduction strategies.
- Development of remediation best practices and standards for indoor environments contaminated with PFAS-containing dust.
- Development of safe and effective destructive PFAS disposal technologies for PFAS-containing materials.

Achieving these recommendations will require coordinated effort between the academic, non-profit, public, and private sectors, including scientists, policymakers, environmental health and safety specialists, and members of the ski community who are already working to address the unique environmental challenges posed by PFAS. The ski industry is well-poised to engage in these efforts given its existing work to address fluorinated wax concerns and its inclination to support environmental initiatives [54]. Advances made towards achieving these recommendations in the context of fluorinated ski waxes will be of interest to other industries facing similar challenges with different kinds of PFAS-containing materials.
